# Less Invasive Rotational Acetabular Osteotomy for Hip Dysplasia

**DOI:** 10.1007/s11999-012-2599-6

**Published:** 2012-09-25

**Authors:** Masaaki Maruyama, Shinji Wakabayashi, Keiji Tensho

**Affiliations:** 1 Department of Orthopaedic Surgery, Shinonoi General Hospital, 666-1 Ai, Shinonoi, Nagano 388-8004 Japan; 2 Department of Orthopaedic Surgery, Chushin Matsumoto Hospital, Matsumoto Medical Center, Matsumoto, Nagano Japan; 3 Department of Orthopaedic Surgery, Shinshu University School of Medicine, Matsumoto, Nagano Japan

## Abstract

**Background:**

Broad dissection with a long skin incision and detachment of the gluteus medius muscle performed for rotational acetabular osteotomy (RAO) can result in weakness in abduction strength of the hip. We use a surgical procedure for RAO that minimizes operative invasion of soft tissue and reduces incision length compared with conventional procedures.

**Questions/Purposes:**

We evaluated the clinical results of this less-invasive RAO comparing it with the more-invasive prior procedure with respect to improvement in clinical hip scores and radiographic coverage and overall hip survival after the procedure.

**Methods:**

In this less-invasive exposure, the medial gluteus muscle is retracted to expose the ilium without detachment from the iliac crest. Similarly, the rectus femoris muscle tendon is retracted, not excised. The lateral part of the osteotomized ilium is cut to form the bone graft instead of harvesting it from the outer cortical bone of the ilium. Between 2000 and 2009, 62 patients (71 hips) underwent this procedure. Twenty-eight hips had early-stage osteoarthritis and 43 had advanced-stage osteoarthritis. Mean patient age was 40 years at the time of surgery. We evaluated improvement in hip scores (Merle d’Aubigné-Postel, Japanese Orthopaedic Association) and radiographic appearance (lateral center-edge angle, Sharp’s angle, acetabular head index [AHI]). Kaplan-Meier survivorship analysis was performed. Mean followup was 5 years (range, 2.0–10.4 years).

**Results:**

Clinical hip scores improved postoperatively. On average, lateral center-edge angle, Sharp’s angle, and AHI improved by 38°, 11°, and 42%, respectively. Predicted 10-year survival rates were 100% and 72% for hips with early- and advanced-stage osteoarthritis, respectively.

**Conclusions:**

In hips with early-stage osteoarthritis treated by this less-invasive approach, no progression of osteoarthritis was documented and Trendelenburg gait was avoided. However, further investigation is necessary for hips with advanced-stage osteoarthritis.

**Level of Evidence:**

Level IV, therapeutic study. See Guidelines for Authors for a complete description of levels of evidence.

## Introduction

Although minimally invasive surgery is commonly used in THAs [8, 19, 22], there are only a few reports regarding minimally invasive surgery for acetabular reorientation osteotomy [2, 20, 28, 29]. There are several reports regarding acetabular redirectional osteotomies for hip dysplasia, such as the triple osteotomy [24], dial osteotomy [14], double osteotomy [25], spherical osteotomy [33], rotational osteotomy [18, 26, 27], and periacetabular osteotomy [7], which are recognized standard surgical procedures for early stages of degenerative osteoarthritis. In adult hip disease, acetabular dysplasia can lead to early-onset degenerative joint disease [5, 30, 34]. Development of advanced osteoarthritis in patients with hip dysplasia has been reported to range from 20% to 50% [3, 4, 14]. Rotational acetabular osteotomy (RAO) initially was described by Ninomiya and Tagawa [18, 27], and now is recognized as an effective reconstructive surgical procedure for treatment of early- or advanced-stage osteoarthritis secondary to developmental acetabular dysplasia [36, 37].

However, this procedure as described [18, 26, 27] requires broad dissection of the ilium, ischium, and pubis and detachment of muscle insertions for adequate exposure and to allow for formation of the bone graft from the outer cortical bone of the ilium [1, 36, 37]. The detachment of the gluteus medius muscle might result in substantial weakness in the abduction strength of the hip. There have been few reports [32] evaluating postoperative abductor muscle function after conventional RAO [18, 36]. In addition, the intraarticular osteotomy used in the original RAO [18, 26, 27] left the teardrop (acetabular floor) in its original position and possibly resulted in deprivation of the osteotomized acetabulum from its blood supply, except for that coming from the capsule. The techniques, such as that of Ganz et al. [7], require extensive exposure and soft tissue release with a long skin incision of 25 cm or more [9, 18, 26, 37]. We believe these extensive exposures impair functional results and lead to cosmetically compromising scars [13]. Teratani et al. [28, 29] reported a less-invasive periacetabular osteotomy with a skin incision length of 9 cm for all cases. Although this is an excellent procedure, it is highly technically demanding, especially in obese patients.

In 1984 Ninomiya and Tagawa modified the RAO using a surgical exposure without transtrochanteric osteotomy [18]. However, with this exposure, a Trendelenburg gait was evident after surgery and persisted in several patients. In response Murase et al. [15, 16] developed the modified Ollier`s approach, and Hasegawa et al. [9] introduced RAO using the transtrochanteric approach with a 25-cm skin incision. To further reduce the morbidity of the exposure we developed a less-invasive approach for ROA in 2000 which incorporated a smaller skin incision, trochanteric osteotomy, and no detachment of the gluteus medius from the ilium (Table 1).

**Table 1:** Comparison of conventional RAO with our surgical procedure

Parameter	Conventional RAO	Our procedure
Length of skin incision (cm)	≥ 20–30	10–15
Number of incisions of the fascia lata	Two	One incision or a y-shaped incision just below the skin incision
Transtrochanteric approach (trochanter osteotomy)	No	Yes
Detachment of the medial gluteal muscle from the ilium	Yes, partially, 7- to 10-cm width from the anterior superior iliac spine*	No
Detachment of the rectus femoris muscle	Yes, completely	No
Division of the external short rotator muscles	Yes, completely	None except for the piriformis
Thickness of the osteotomized acetabulum cephalad to the joint space (cm)	1.5*–2.5^†^ (1* to 1.5^†^ finger breadths)	2.5 (1.5 finger breadths)
Bone graft for gap between the osteotomized acetabulum and the ilium	Yes; one or two trapezoidal bone grafts from the outer table of the iliac wing	Yes; one trapezoidal bone graft from the lateral part of the osteotomized acetabulum

* From Ninomiya and Tagawa [18]; ^†^from Yasunaga et al. [37]; RAO = rotational acetabular osteotomy.

The purpose of our study was to describe this less-invasive surgical technique and report on the improvements in clinical hip scores, noting especially the presence or absence of a Trendelenburg gait. Our second aim was to assess the reconstruction radiographically, and third, to assess hip survivorship after the procedure. Finally, we compared this new approach with the more-invasive exposure using historical records and patients having the prior exposure on the contralateral side.

## Patients and Methods

We performed the modified RAO in 66 patients (75 hips) between August 2000 and April 2009. Sixty-one patients were female, and five were male (Table 2). The mean ± SD age of the patients at the time of surgery was 39.7 ± 10.3 years (range, 19–65 years). Before being indicated for the procedure, all patients had preoperative AP view radiographs in supine, standing, and abducted positions and a false-profile view of the affected hip in flexion. Osteoarthritis of the hip was classified according to the radiographic findings with the modified system of Ninomiya [17]: Stages 1 and 2 indicate no or slight narrowing of the joint space associated with sclerosis of the subchondral bone (30 hips); Stage 3 indicates marked narrowing of the joint space associated with cystic lucencies and small osteophytes in the femoral head and acetabulum (34 hips); and Stage 4 indicates a joint space less than 1 mm with marked osteophyte formation (11 hips). Radiographs of patients with Stages 3 and 4 osteoarthritis were examined for femoral head deformity, a finding that could result in disruption of the congruency of the hip. Joint space width was evaluated on the standing radiograph. We regarded Stages 1 and 2 as early-stage osteoarthritis and Stages 3 and 4 as advanced-stage osteoarthritis [9]. Surgical indication for the modified RAO included symptomatic acetabular dysplasia with an improvement in joint congruency observed on the AP radiograph with the hip in abduction [18, 36] and a lateral center-edge (CE) angle less than 16° [35]. In addition, patients with more advanced-stage osteoarthritis (Stage 4) were included only if they had less than 1 mm narrowing of the joint space or partial disappearance observed on standing AP radiographs but with emergence of the joint space in the hip abducted position. All 75 hips underwent RAO, and two of the hips with Stage 4 osteoarthritis underwent a concomitant proximal femoral valgus osteotomy. Four patients (four hips) were lost to followup: two with early-stage ostearthritis and two (one Stage 3; one Stage 4) with advanced-stage osteoarthritis. This left 62 patients (71 hips) available for followup. Minimum followup was 2.0 years (mean ± SD, 5.3 ± 2.7 years; range, 2.0–10.4 years). All study patients gave informed consent before surgery.

**Table 2:** Patient demographics and results

Variable	All patients
Number of patients/hips	66/75
Female:male (number of patients)	61:5
Age at time of surgery (years)*	39.7 ± 10.3 (19–65)
Height (cm)*	157.2 ± 6.7 (140.9–169.5)
Weight (kg)*	56.5 ± 10.0 (38.0–83.0)
BMI*	22.9 ± 3.9 (16.3–31.8)
Osteoarthritis stage (number of hips)	
1 + 2	30
3	34
4	11
Followup (years)*	5.3 ± 2.7 (2.0–10.4)

* Values are expressed as mean ± SD, with range in parentheses.

**Fig. 1:**
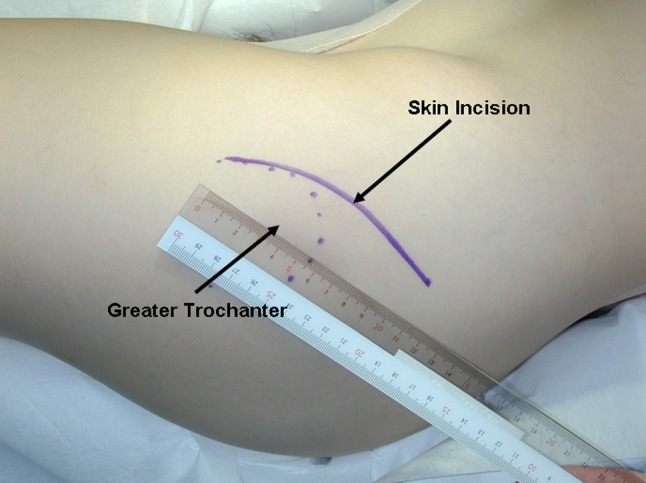
The skin incision begins distally in the anterodistal border of the greater trochanter and extends proximally via a curved line over the trochanter.

**Fig. 2:**
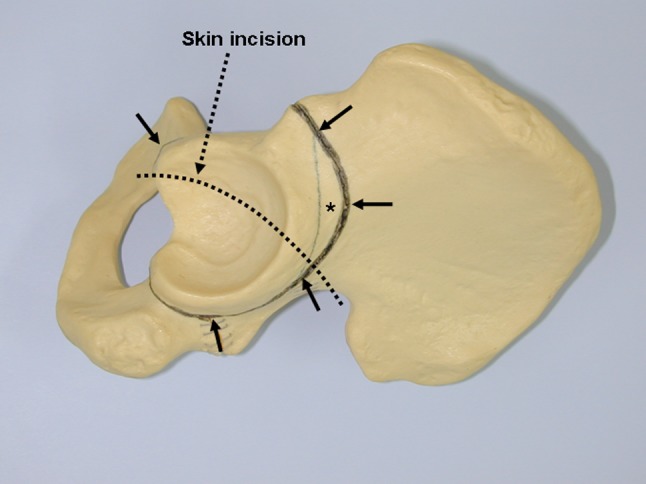
The osteotomy line (arrows) is shown on the left pelvis. The thickness of the osteotomized acetabulum is approximately 2.5 cm to the joint space at the proximal (cephalad) portion. A lunate (lateral view) and trapezoid (AP view) shaped bone graft (asterisk) can be obtained from the lateral part of the osteotomized fragment.

**Fig. 3:**
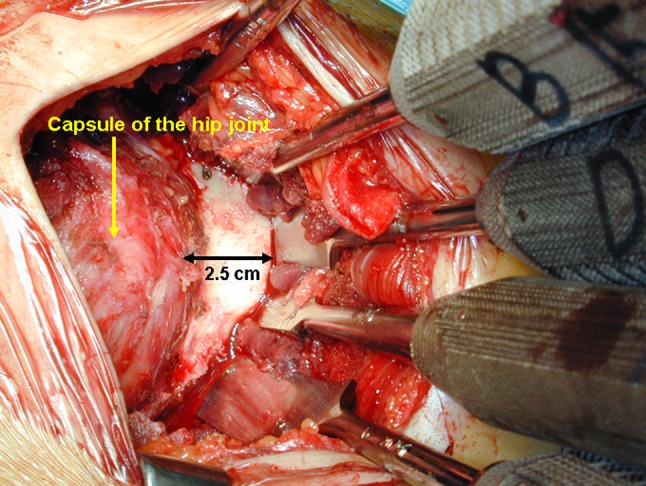
The osteotomy line lies approximately 2.5 cm (1.5 finger breadths) cephalad to the joint space (left side).

**Fig. 4:**
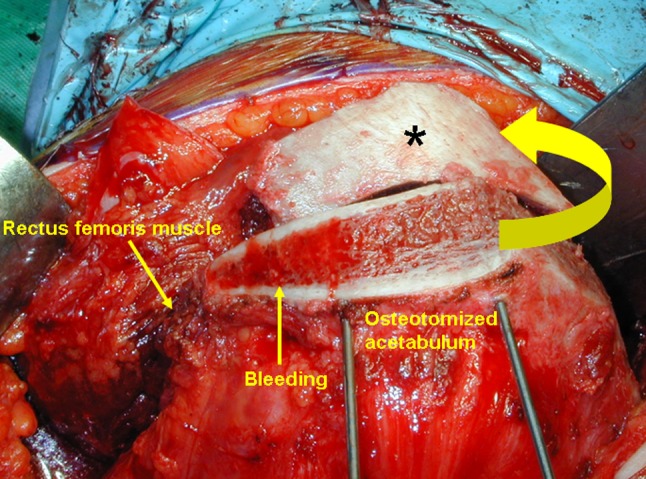
A bone graft from the lateral part of the osteotomized acetabulum (asterisk) is shifted (curved arrow) and transfixed by Kirschner wires (left hip). Bleeding from the anterior part of the osteotomized acetabulum indicates blood supply from the rectus femoris muscle.

**Fig. 5:**
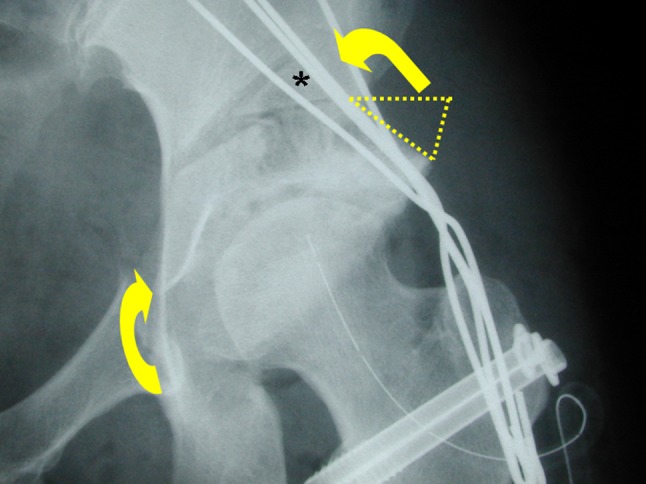
An AP postoperative radiograph of the left hip shows the osteotomized acetabulum is rotated anterolaterally (lower arrow) with a trapezoid-shaped bone graft (asterisk) from the lateral part of the osteotomized fragment (upper arrow).

To perform the procedure, the patient was placed in the lateral decubitus position and stabilized on an air-fluidized body fixation system (Magic bed™, Nikko Fines Industries Co Ltd, Tokyo, Japan [same as the Vac-Pac™ Surgical Positioning System; Olympic Medical Co Ltd, Seattle, WA, USA]). Fluoroscopy confirmed the site and location of the osteotomy for chisel placement and assisted in evaluating the acetabular reduction and fixation intraoperatively. A direct lateral approach to the hip was used for exposure. The skin incision began distally in the anterodistal border of the greater trochanter and extended proximally via a curved line over the trochanter (Fig. 1). The total length of the incision ranged from 10 to 15 cm, depending on the thickness of the subcutaneous fat and depth to the acetabulum. The fascia lata often was incised using a y shape along the anterior border of the gluteus medius muscle to expose the anterior inferior iliac spine. It was unnecessary to incise the superficial aponeurosis of the tensor fasciae latae, which was left intact to protect the lateral cutaneous femoral nerve during surgery. An osteotomy of the greater trochanter was done by using a 3-cm-wide chisel and the trochanter was proximally reflected with the gluteus medius and minimus muscles. The rectus femoris muscle was not detached from the anterior inferior iliac spine. The ilium (acetabulum) was exposed by retraction of the gluteus medius muscle without detachment from its origin on the ilium and iliac crest. The short rotator muscles and their tendons, except for the piriformis, were not released but rather retracted distally during the ischial osteotomy. The gluteus minimus muscle was separated from the capsule of the hip by blunt dissection. Then, the gluteus medius and minimus muscles were reflected proximally from the joint space or the acetabular rim and held with large custom-made retractors during acetabular osteotomy. The ilium and ischium were spherically cut in continuity. The osteotomy line (Fig. 2) was started just proximal to the inferior anterior iliac spine and passed through a point approximately 2.5 cm proximal (approximately 1.5 finger breadths) from the joint space (Fig. 3). The proximal thickness of the osteotomized acetabulum was at least 1.5 cm because Trousdale et al. [31] and Hasegawa et al. [9] reported that osteonecrosis of the osteotomized acetabulum might occur if thickness of the acetabulum was too thin. Then, the line was passed through the midpoint between the posterior acetabular ridge and the greater sciatic notch and ended at the innominate sulcus of the ischium. The osteotomy was performed by using a specially curved chisel introduced by Ninomiya and Tagawa [18, 26, 27] after decortication using a straight chisel. Osteotomy of corpus of the pubis and the iliopectineal ridge were performed under careful palpation and image intensification, as they could not be observed directly during the surgical procedure. We used a special wooden hammer introduced by Murase et al. [15, 16] to listen carefully to the sound of the hammer to detect when the tip of the osteotome had reached the inner cortex of the ilium and to avoid excessive penetration into the intrapelvic space. Visible bleeding indicative of a good blood supply often was observed from the anterior part of the osteotomized acetabulum where the rectus femoris muscle was attached (Fig. 4). If necessary, the lateral part of the osteotomized ilium (acetabulum) was cut in a lunate (lateral view) and a trapezoid (AP view) shape to fashion the bone graft instead of using the outer cortical table of the iliac wing (Fig. 5). The size of the graft was estimated based on preoperative planning and measurement. After fluoroscopic confirmation of adequate coverage of the femoral head by the rotated acetabulum and the medially shifted femoral head, several Kirschner wires, 1.8 mm in diameter, were used to transfix the osteotomized acetabulum and the lunate-form bone graft to the pelvis. The operative field was irrigated with saline solution and the osteotomized greater trochanter was repositioned and fixed with two cannulated cancellous hip (CCH) screws. For the patients with abductor weakness including the two patients with a concomitant proximal femoral valgus osteotomy, distal transposition of the greater trochanter was performed simultaneously to gain effective excursion of the medial gluteus muscle. The indication for the valgus osteotomy was based on the description by Ninomiya and Tagawa [18].

Warfarin was used routinely for deep venous thrombosis prophylaxis, and a few patients recognized as being at higher risk were administered low-molecular-weight heparin for 2 weeks.

Cast immobilization was not used, but postoperative traction was maintained for 10 days of bed rest to avoid excessive stress to the rotated acetabular fragment. Patients were allowed to use a wheelchair with touch-down weightbearing after passive and assisted active ROM exercises as instructed by our physiotherapists. Partial weightbearing ambulation with the aid of crutches was begun 5 to 6 weeks after surgery: 1/6 body weight on the affected leg for 6 to 8 weeks followed by an increasing weightbearing program supervised by the physiotherapists under the guidance of the operating surgeon (MM). Hardware, such as Kirschner wires and CCH screws, routinely were removed 7 weeks postoperatively.

Clinical followup was performed semiannually using the Merle d’Aubigné-Postel system [6] and the Japanese Orthopaedic Association (JOA) hip score [10] with evaluation of ambulation for the presence of a Trendelenburg gait by the operating surgeon (MM) and his colleagues. The hips were assessed radiographically to evaluate correction of deformity, measuring the lateral center-edge (CE) angle of Wiberg [35], Sharp’s angle [23], and acetabular head index (AHI) of Heyman and Herndon [11] preoperatively and postoperatively. One of us (MM) performed the radiographic measurements using a digital caliper (Mitsutoyo, Tokyo, Japan) with an accuracy of ± 0.02 mm. Healing of the osteotomy site and progression of osteoarthritis also were assessed on postoperative radiographs semiannually.

We used 2010 version SSRI^®^ software (SSRI Inc, Tokyo, Japan) to perform the statistical analysis and Excel^®^ (Microsoft Corp, Redmond, WA, USA) for descriptive statistics. Paired t-tests were used to compare radiographic parameters (lateral CE angle, Sharp’s angle, AHI) and clinical hip scores (Merle d’Aubigné-Postel, JOA) between preoperative and postoperative groups. We performed Kaplan-Meier survivorship analysis to estimate the probability of progression and change of the radiographic osteoarthritis stage. The association of preoperative joint disease with postoperative clinical scores less than those preoperatively and radiographic signs of progression of osteoarthritis as the end point was assessed with the log-rank test. The 95% CIs were calculated. Ten patients in this series with previous surgery on the contralateral hip by the conventional surgical procedure developed by Ninomiya and Tagawa [18, 26] or by the modified Ollier`s transtrochanteric approach [15, 16] with partial detachment of the gluteus medius muscle from the ilium were analyzed using Fisher’s exact test and the Mantel-Haenszel test. For all analyses, statistical significance was defined as p values less than 0.05.

## Results

The average operative scar lengths measured at latest followup were 17.3 ± 2.2 cm (range, 15.5–21 cm) for 33 hips operated on between August 2000 and August 2004 and 12.9 ± 1.4 cm (range, 10–15 cm) for 38 hips operated on between September 2004 and April 2009. The length of the skin incision was gradually shortened secondary to the surgeon becoming more proficient in the surgical procedure. There were no major neurovascular injuries or intraarticular penetration of the osteotome intraoperatively.

Clinical hip scores improved in all patients from preoperatively to postoperatively (Table 3). In the hips with early-stage osteoarthritis, the average Merle d’Aubigné-Postel score improved (p < 0.001) from 11.1 ± 1.7 (range, 6–14) preoperatively to 17.8 ± 0.2 (range, 17–18) at latest followup. JOA hip scores also improved (p < 0.001) from 58.4 ± 9.9 (range, 30–78) to 98.2 ± 1.8 (range, 95–100). In the hips with advanced-stage osteoarthritis, the average Merle d’Aubigné-Postel score improved (p < 0.001) from 10.6 ± 1.6 (range, 7–13) preoperatively to 15.4 ± 2.7 (range, 8–18) at latest followup. JOA hip scores also improved (p < 0.001) from 56.0 ± 9.1 (range, 31–74) to 87.1 ± 14.6 (range, 43–100).

**Table 3:** Clinical results after RAO

Variable	Early-stage osteoarthritis			Advanced-stage osteoarthritis		
	Preoperative*	Postoperative*	p value	Preoperative*	Postoperative*	p value
Merle d’Aubigné-Postel score (points)	11.1 ± 1.7 (6–14)	17.8 ± 0.2 (17–18)	< 0.001	10.6 ± 1.6 (7–13)	15.4 ± 2.7 (8–18)	< 0.001
JOA hip score (points)	58.4 ± 9.9 (30–78)	98.2 ± 1.8 (95–100)	< 0.001	56.0 ± 9.1 (31–74)	87.1 ± 14.6 (43–100)	< 0.001

* Values are expressed as mean ± SD, with range in parentheses; RAO = rotational acetabular osteotomy; JOA = Japanese Orthopaedic Association.

Comparison of preoperative and followup radiographs for all patients showed an improvement in deformity (Table 4). The lateral CE angle of Wiberg improved (p < 0.001) on average by 37.8° from −1.3° ± 10.1° (range, −44° to 15°) preoperatively to 36.5° ± 8.9° (range, 15°–52°) at latest followup. Sharp’s angle improved (p < 0.001) on average by 10.9° with a reduction from 50.3° ± 4.3° (range, 40°–64°) to 39.4° ± 4.8° (range, 26°–50°). The average improvement in AHI was 41.7%, from 54.0% ± 10.7% (range, 13%–85%) to 95.7% ± 7.9% (range, 81%–113%) (p < 0.001).

**Table 4:** Radiographic results after RAO

Variable	All patients			p value
	Preoperative*	Postoperative*	Difference	
Lateral CE angle (°)	−1.3 ± 10.1 (−44 to 15)	36.5 ± 8.9 (15–52)	37.8	< 0.001
Sharp’s angle (°)	50.3 ± 4.3 (40–64)	39.4 ± 4.8 (26–50)	10.9	< 0.001
AHI (%)	54.0 ± 10.7 (13–85)	95.7 ± 7.9 (81–113)	41.7	< 0.001

* Values are expressed as mean ± SD, with range in parentheses; RAO = rotational acetabular osteotomy; CE = center-edge; AHI = acetabular head index.

**Fig. 6A–B:**
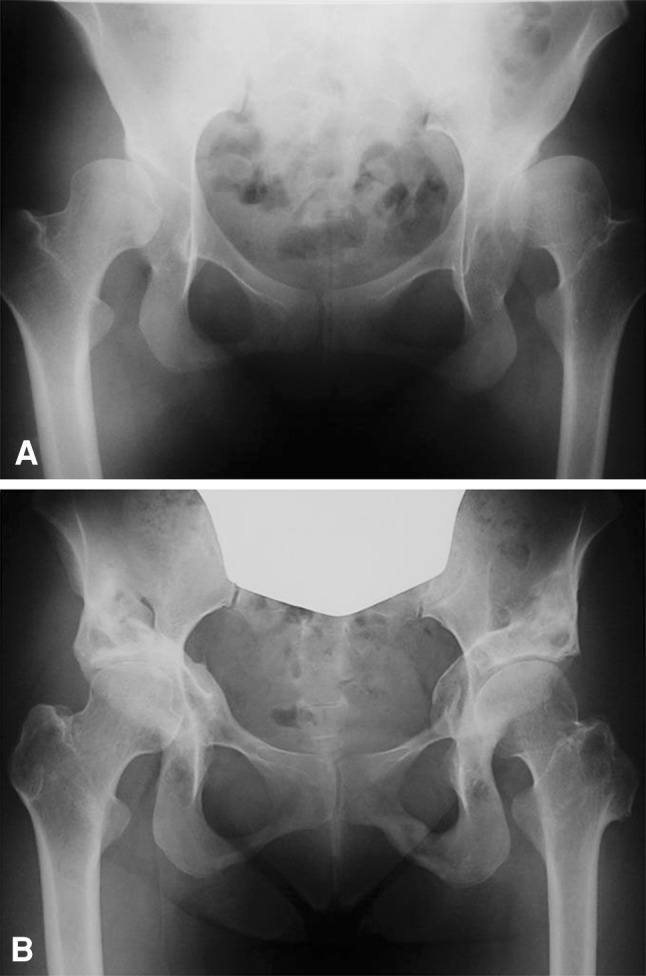
(**A**) A preoperative AP radiograph of the hips of a 19-year-old girl (the same patient whose radiograph is shown in Fig. 5) shows early-stage dysplastic osteoarthritis for the right hip and advanced-stage for the left hip. The right and left lateral CE angles are −13° and −44°, respectively. (**B**) Five years after right RAO and 7 years after left RAO, the right and left lateral CE angles are 33° and 20°, respectively. Both femoral heads were shifted medially and distally, and the left greater trochanter was pulled down and reattached. There was no progression of osteoarthritis.

Kaplan-Meier survivorship analysis predicted a 10-year survival rate of 100% for hips with early-stage osteoarthritis and 72.1% ± 7.8% (95% CI, 56.8%–87.4%) for hips with advanced-stage osteoarthritis. All acetabular osteotomy sites healed within 1 year postoperatively, and remodeling and trabecular reorientation of the osteotomized acetabulum occurred within 3 years postoperatively even in cases with severe dysplasia (Fig. 6). In this case, the current RAO technique also achieved reduction of horizontal offset postoperatively. Indications of the rotational osteotomy mostly overlapped those of redirectional osteotomy. The difference between these osteotomies was that the former was used for more severe dysplastic hips with lateral CE angles less than −30° (Fig. 6). Complications occurring during or after surgery were transient paresis of the lateral femoral cutaneous nerve in two patients and ectopic bone formation in 15 patients. In one of the latter patients, an additional operation for extraction of the ectopic bone was performed 1.5 years after the primary RAO. The osteotomized acetabulum and reattached greater trochanter healed in all hips. No progression of osteoarthritis was observed in the hips with early-stage osteoarthritis without Trendelenburg gait. Ten hips with advanced-stage osteoarthritis (two in Stage 3, eight in Stage 4) preoperatively had radiographic evidence of progression of osteoarthritis with Trendelenburg gait, and six of them subsequently underwent THA between 2 to 4 years after RAO.

All 10 patients who had undergone previous surgery in the contralateral hip were more satisfied with the cosmetic appearance and scar length with the current procedure (mean length, 13.5 cm) than with the previous scar length (mean length, 26.5 cm). No Trendelenburg gait was observed in patients who underwent the current procedure but four patients (four hips) who had the previous technique had a Trendelenburg gait (Fisher’s exact test, p < 0.05; Mantel-Haenszel test, p < 0.03).

## Discussion

Broad dissection with a long skin incision and detachment of the gluteus medius muscle performed for RAO can result in weakness in the abduction strength of the hip. We aimed to reduce surgical invasion of conventional acetabular osteotomies by developing a modified approach and technique. The advantages of the new technique are as follows: (1) one approach with a relatively short skin incision is used, which provides enough exposure by osteotomizing the greater trochanter; (2) the muscle power of the hip abductor is preserved to prevent a postoperative Trendelenburg gait by retraction of the medial gluteus muscle without detachment from the ilium and iliac crest; (3) blood supply from the rectus femoris muscle to the osteotomized acetabulum is preserved as the rectus femoris tendon is not detached from the anterior inferior iliac spine, and blood supply to the capsule also is preserved as the osteotomy is extraarticular, including the acetabular floor (ie, teardrop in radiograph); and (4) if necessary, the lateral part of the osteotomized ilium (acetabulum) is cut in lunate (lateral view) and trapezoid (AP view) form for the bone graft instead of the outer cortical bone of the ilium to avoid excessive exposure. A fifth possible advantage of this procedure that was not evaluated in this study is that the shape of the pelvis, especially the pelvic ring, is unchanged, permitting normal vaginal delivery, a factor of importance, as most patients with acetabular dysplasia are adolescent and young women. By using this procedure, the patients in our series achieved good clinical hip scores and hip survivorship with reduction of postoperative Trendelenburg gait.

However, this study has several limitations including a relatively short followup, small numbers of subjects especially for comparison to conventional RAO, and potential observer bias.

The total length of the skin incision was 25 cm [9] for the bikini incision and conventional RAO. In the case of an ilioinguinal incision through a modified Smith-Petersen approach, such as a Bernese periacetabular osteotomy (Ganz), the skin incision length was at least 20 cm or more [7, 13, 21]. The advantages of trochanteric osteotomy are that it allows a visible continuous osteotomy line in an operative field, and distal transposition (ie, pulling down) of the greater trochanter is available to control tension of the hip abductor muscle. A disadvantage is that it takes one more procedure for reattachment of the greater trochanter. Our surgical procedure was not minimally invasive because muscle resection of the gluteus minimus and short rotator (piriformis) were inevitable, although these resections and reattachments do not influence abductor strength. As this is one limitation of our study, we called this procedure less invasive.

The predicted 10-year survival rate in this study was 72.1% for advanced-stage osteoarthritis hips, which is similar to rates of 72.2% after 10 years [36], 79% after 11 years [12], and 71% after 8 years [8] in other reports. In addition, the affected hips functioned well clinically without weakening the hip abductor muscles in almost all cases. Thus, it seems there is no disadvantage of this less-invasive procedure in patients with Stages 1 to 3 osteoarthritis. However, on the basis of our data, this type of osteotomy is not recommended for patients with more advanced-stage osteoarthritis (Stage 4) who had aggravation of joint incongruency, narrowing of the joint space less than 1 mm observed on AP radiographs of the patient in the standing position, and especially partial disappearance (Class D congruency according to Yasunaga et al. [37]) of the cartilage space in the hip abducted position.

Our approach requires a single skin incision of only 10- to 15-cm length, which provides satisfactory exposure by only retracting the gluteus medius with an osteotomized greater trochanter and an approach only from the outside of the ilium with a osteotome. Although this surgical technique might provide a benefit to the patients undergoing RAO, especially for cosmesis, which is important to younger female patients, further investigation is necessary to examine the benefit relative to long-term clinical and radiographic results.

Our surgical procedure focused on a short skin incision, retraction (not excision) of the gluteus medius muscle with an osteotomized greater trochanter, no excision of the rectus femoris muscle, and an approach only from the outside of the ilium with a osteotome. We had no patients with Trendelenburg gait postoperatively and good clinical results without progression of osteoarthritis in hips with early-stage ostearthritis. However, further investigation is necessary for hips with advanced-stage osteoarthritis.
